# Case Report: Intramural colonic signet ring cell carcinoma presenting as intestinal pseudo-obstruction: A case presentation and review of the literature

**DOI:** 10.3389/fonc.2023.1059368

**Published:** 2023-03-23

**Authors:** Yuxia Li, Genmei He, Ruqin Zhong, Xuejuan Li, Huamei Li, Huaqiong Dong, Yun Zhang, Guohong Zhao, Leilei Fang

**Affiliations:** ^1^ Department of Gastroenterology, Shanghai Tenth People’s Hospital, School of Medicine, Tongji University, Shanghai, China; ^2^ Department of Gastroenterology, People’s Hospital, Lahu-Wa-Bulang-Dai Autonomous County of Shuangjiang, Lincang, Yunnan, China; ^3^ Kunming Jinyu Medical Laboratory Co., Ltd, Yunnan, China

**Keywords:** signet ring cell carcinoma, colorectal cancer, incomplete bowel obstruction, laparoscopicright hemicolon carcinoma radical resection, prognosis

## Abstract

Colorectal cancer (CRC) is the third most common cancer in the world. Other than adenocarcinomas, exceptional tumors of the colon and rectum represent a neglected clinical issue due to their rarity. Signet ring cell carcinoma (SRCC) is a rare subtype of CRC and has an extremely poor prognosis due to its advanced stage at diagnosis. Here we report a rare case of colorectal SRCC manifested as recurrent intestinal obstruction with a negative colonoscopy. Finally, he was diagnosed with signet ring cell carcinoma of the colon by postoperative pathology. It emphasized the special feature of intramural tumor growth without penetrating the mucosa in SRCC, which requires timely surgical intervention to avoid delay in diagnosis and treatment.

## Introduction

Colorectal cancer (CRC) is the third most common cancer in the world and ranks as the second leading cause of cancer-specific death globally (1). Adenocarcinoma (AC) represents the most frequent form of CRC, accounting for about 98% of tumor histological types. However, other than adenocarcinomas, exceptional tumors of the colon and rectum represent a neglected clinical issue due to their rarity.

Primary signet-ring cell carcinoma (SRCC) is more likely to be seen in the stomach, which has been the fourth most common cause of cancer-related mortality worldwide (2). Colorectal signet ring cell carcinoma (SRCC) is rare and only accounts for about 1% of CRC subtypes with poor prognosis (3). It has the typical appearance of a “signet ring” because the nuclei are pushed to the periphery of the cell by the intracytoplasmic mucinous component. Formally, a tumor is labeled SRCC when >50% of tumor cells show a “signet ring,” whereas ACs with <50% signet ring cells are still classified as ACs with a signet ring cell component (4). Different from AC with morphology of intraluminal mass, colorectal SRCC often presents a markedly narrowed lumen due to diffuse circumferential thickening of the bowel wall and sometimes has an association with inflammatory bowel diseases (5, 6). Previous studies analyzed the primary symptoms of patients with colorectal SRCC at diagnosis and suggested its special features, like atypical and delayed clinical manifestations, a younger age at onset, and high false-negative rates of endoscopic biopsy, may result in frequently advanced stages and a poor prognosis in colorectal SRCC (7–9). In CRC, SRCC is generally considered to be associated with microsatellite instability-high (MSI-H), a well-established prognostic biomarker for better survival in patients with localized tumor stages (10). However, signet-ring cell carcinoma is associated with shorter survival in CRC patients, moreover, SRCC is associated with higher mortality even when the signet-ring cell component is less than 50% (10, 11). The therapy recommended for colorectal SRCC is typically palliative chemotherapy, which is identical to that recommended for AC (12).

In this case, we reported that a 54-year-old man who developed repeated bowel obstruction with a negative endoscopic biopsy was finally diagnosed with colorectal SRCC by postoperative histopathology. It emphasized the special feature of intramural tumor growth without penetrating the mucosa in SRCC, which required multiple examinations and timely surgery in case of a delay in diagnosis.

## Case presentation

A 54-year-old man was admitted to the People’s Hospital, Lahu-Wa-Bulang-Dai Autonomous County of Shuangjiang, Lincang, Yunnan, China, complaining of abdominal pain, diarrhea, nausea, and vomiting for more than 1 month. He had presented abdominal pain accompanied by yellow, watery diarrhea (two to three times a day) intermittently for the past month, and vomiting could relieve these symptoms at an early stage. However, his symptoms of abdominal pain and vomiting have been getting worse gradually over the last week. Thus, he was hospitalized at the People’s Hospital of Shuangjiang on 12 August 2021.

**Figure 1:**
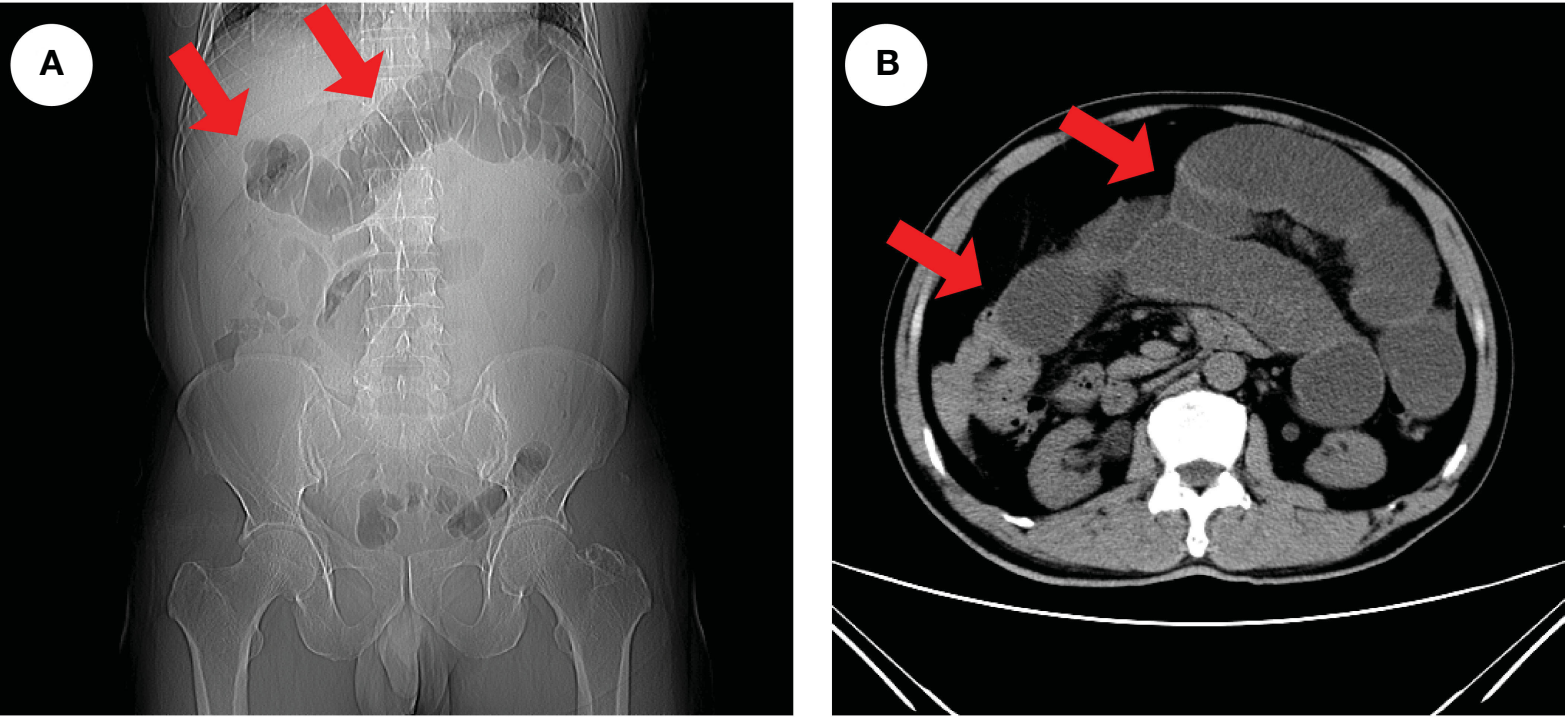
Radiologic findings of intestinal obstruction. An abdominal CT examination in an emergency was performed on the patient and revealed extensive dilatation of the transverse colon (red arrows), indicating proximal intestinal obstruction **(A, B)**.

**Figure 2:**
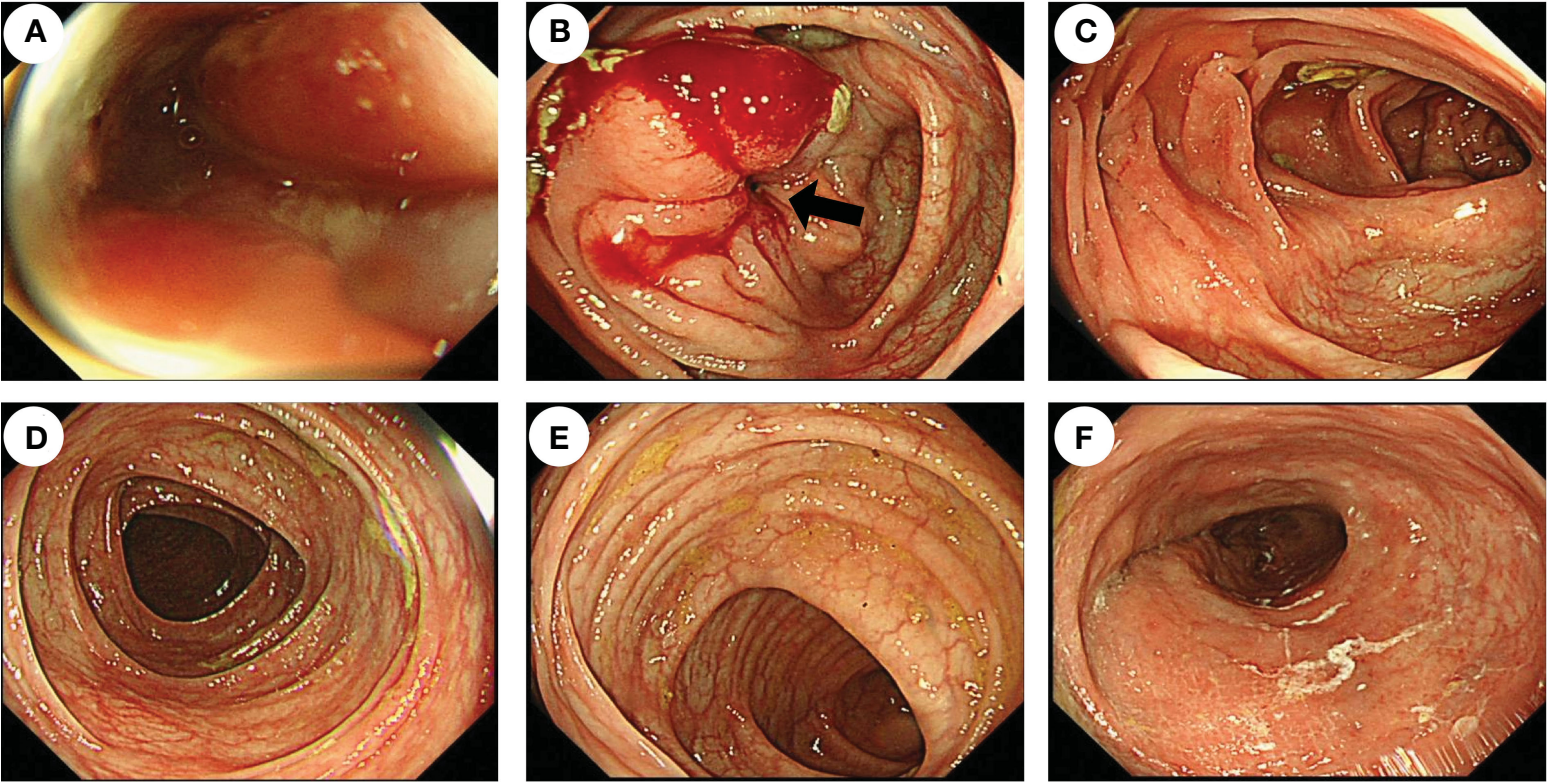
Endoscopic features of the patient before surgery. Colonoscopy showed that the cavity was obviously narrow in the ileocecal region (black arrow), which made the endoscope unable to pass through **(A, B)**, but there were no obvious abnormalities in the ascending colon **(C)**, transverse colon **(D)**, descending colon **(E)**, or rectum **(F)**.

**Figure 3:**
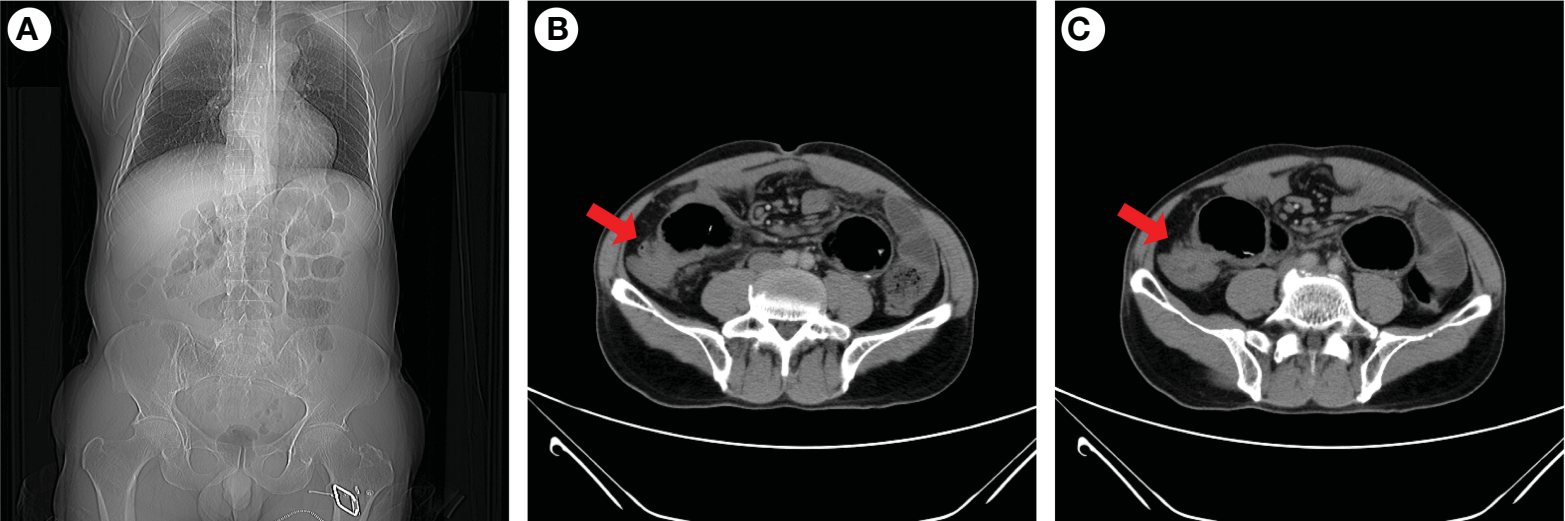
Abdominal CT examination of the patient when intestinal obstruction occurs again. Coronal images indicated gas and fluid accumulation in the intestine **(A)**, and transverse images revealed ileocecal intestinal wall thickening **(B, C)**. The red arrow indicaes the ileocecal junction.

**Figure 4:**
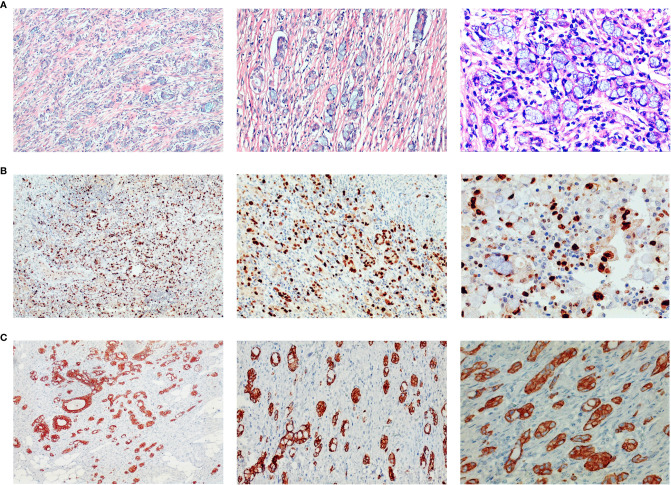
Histopathology of surgical specimens. Postoperative pathology showed signet ring cell carcinoma among moderately-to-poorly differentiated AC in the right colon. Hematoxylin and eosin staining showed signet-ring cell carcinoma **(A)**, and further immunohistochemistry staining showed ki-67 (>80% +) **(B)**, and CK8 (+) **(C)**. Pictures were magnified ×100 on the left, ×200 in the middle, and ×400 on the right.

**Figure 5:**
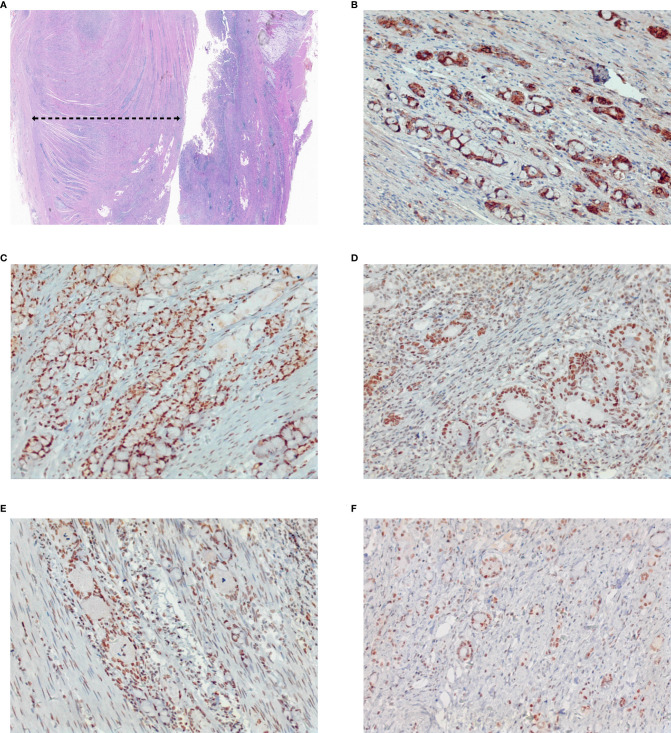
Pathological aggressiveness and microsatellite instability of the tumor. The tumor occupies about 80% of the whole tumor **(A)**. Immunohistochemistry staining showed E-Cadherin (+) **(B)**, MLH1 (+) **(C)**, MSH2 (+) **(D)**, MSH6 (+) **(E)**, and PMS2 (+) **(F)**, which indicates it is microsatellite instability-low (MSI-L) cancer. Original magnification ×4 for **(A)**, and magnification ×200 for **(B–F)**.

He is nondrinking, nonsmoking, and has a history of renal insufficiency (serum creatinine level: 173.1 umol/L, n.v., 50–90 umol/L) due to renal calculus for 2 years. He has no family history of cancer or other gene-related diseases. On admission, a physical examination showed mild tenderness in the whole belly and a fading gurgling sound. Routine blood tests, coagulation function, hepatitis B, HIV, syphilis, liver function, myocardial enzymes, electrolytes, blood lipids, and glucose were all normal. Laboratory tests revealed slight increase in squamous cell carcinoma (SCC, 4.79 ng/ml; normal value (n.v.), <2.5 ng/ml), but carcinoembryonic antigen (CEA, 0.962 ng/ml; n.v., <5.093 ng/ml), carbohydrate antigen (CA)-199 (6.82 IU/ml, n.v., <37 IU/ml), CA-50 (2.24 IU/ml; n.v., <25 IU/ml), CA-242 (2.09 IU/ml; n.v., <20 IU/ml), CA-724 (1.77 IU/ml; n.v., <6 IU/ml), alpha-fetoprotein (AFP, 1.61 IU/ml; n.v., <6.05 IU/ml), neuron-specific enolase (NSE, 2.51 ng/mL; n.v., <6 ng/ml), abdominal computed tomography (CT) examination in emergency was performed to him and suggested intestinal obstruction (**Figures 1A, B**). As he presented no fever or chill and maintained diarrhea (three to four times a day), we provided conservative treatment after getting informed consent. Then he was given fasting, gastrointestinal decompression, empirical anti-infection therapy (ampicillin, 4 g, q12 h), and nutritional support for 5 days. When his obstructive symptoms were relieved, he underwent gastroscopy and colonoscopy during hospitalization. Gastroscopy showed no obvious abnormalities, and colonoscopy suggested ileocecal intraluminal stricture (**Figures 2A–F**), which made us unable to observe the end of the ileum. We recommended further CT enterography for this patient, but he refused this examination due to the potential risk of recurrent intestinal obstruction. No evidence of vasculitis, fungal elements, tuberculosis, or parasitic infection was found in further laboratory tests. Endoscopic biopsies of the ileocecal region revealed negative histopathology. Thus, he was discharged from the hospital on 20 August 2021, and it was suggested that he have a liquid diet for at least two weeks and be followed up in an outpatient clinic every month. However, he presented with abdominal pain and diarrhea (six to eight times a day) again in 1 month, and it was even worse than before. Immediately, he was admitted to the Department of Emergency at the People’s Hospital of Shuangjiang and received an abdominal CT examination on 24 September 2021. CT examination revealed a thicker intestinal wall than normal in the ileocecal region, indicating diverticulitis or tumor (**Figures 3A–C**). Radiographic examinations of the head, chest, and abdomen excluded distant metastases. Therefore, the patient received laparoscopic right hemicolon carcinoma radical resection in the Department of Surgery on 29 September 2021. Postoperative pathology revealed that it is a signet ring cell carcinoma with a size of 3 × 2.5 cm among moderately-to-poorly differentiated AC in the right colon (**Figures 4A–C**). SRCC occupies about 80% of the whole tumor with no heterogeneity (**Figure 5A**). The tumor invaded the whole layer of the colonic wall and partly nerves. Tumor thrombus can be found in vessels. No cancer invasion was found along the cutting edges of the surgical specimen. Immunohistochemistry staining showed E-Cadherin (+), MLH1 (+), MSH2 (+), MSH6 (+), and PMS2 (+), which indicates it is MSL-low (MSI-L) cancer (**Figures 5B–F**). Metastasis was found in the pericolonic lymph nodes (5/17). Afterwards, he was given regular chemotherapy with the FOLFOX strategy. He received a reexamination, including a colonoscopy and abdominal CT, on 8 February 2022, which revealed no obvious abnormalities. The patient was followed up in the Department of Gastroenterology at the People’s Hospital of Shuangjiang until the completion of the article (July 2022) with a good prognosis.

## Discussion

Colorectal SRCC is a histologically rare subtype of colorectal cancer (~1%) with atypical clinical manifestations, different pathological features, and indistinguishable biological behaviors compared to AC (13, 14). Unlike the intraluminal mass in AC, the patient with colorectal SRCC in this case appears to have thickening of the bowel wall in the right colon with a markedly narrowed lumen, which was suspected to be inflammatory bowel disease in the first place. As his symptoms of intestinal obstruction gradually worsened, he received an operation and was finally diagnosed with colorectal SRCC with AC according to postoperative pathology at the age of 54. Previous population-based studies revealed a mean age of about 65 years old at onset for colorectal SRCC, which is 3.5 years earlier than that of AC (3). However, the patient in this case was diagnosed much younger (54 years old) than the mean age (65 years old) for colorectal SRCC, which reminds clinical physicians to be on alert for colorectal SRCC with atypical features including a younger age at onset, clinical manifestations of incomplete intestinal obstruction, and negative results of endoscopy (6). Surgical treatment is necessary for patients with recurrent incomplete intestinal obstruction at a relatively young age.

The overall prognosis of colorectal SRCC is extremely poor, which may be due to the advanced stage at diagnosis (15, 16). SRCC was associated with worse 5-year survival significantly compared with AC in a population-based study including 1,972 colorectal SRCC cases. The survival difference was prominent at stage III (17). There were also various studies reporting that SRCC histology is an independent adverse prognostic factor after adjustment for covariates including tumor stage and location (10, 18). In CRC, MSI-H, or mismatch repair deficient (dMMR), is a well-established prognostic biomarker for better survival in SRCC patients with localized cancer stages, but SRCC is associated with shorter survival compared with other CRC patients (8, 10). It was reported that undefined postoperative adjuvant chemotherapy improved survival time in 936 stage II–III patients (19). The patient in this case underwent routine chemotherapy postoperatively according to a previous report that SRCC benefitted comparably from adjuvant fluorouracil-based chemotherapy compared with AC (17). He survived for 10 months until the time when this article finished with a good prognosis. We will continue to follow up in the future. It emphasized the special feature of intramural tumor growth without penetrating the mucosa in SRCC, which requires prompt operation in case of a delay in diagnosis. It gives us a good lesson that clinicians need to be highly alert to the occurrence of rare tumors in patients with recurrent episodes of intestinal obstruction with negative endoscopy. Meanwhile, it is necessary to consider surgical intervention in time and pay close attention to postoperative pathology.

In conclusion, this article reported a rare case of colorectal SRCC manifested as recurrent bowel obstruction and a negative result of an endoscopic biopsy. It emphasized the special feature of intramural tumor growth without penetrating the mucosa in SRCC, which requires timely surgical intervention to avoid delay in diagnosis and treatment. Postoperative treatment with fluorouracil-based chemotherapy may improve patients’ survival time. The black dotted line refers to the tumor area.

## Limitation

As a result of the patient’s financial situation, there are no images of the gross specimen after surgery, nor are there positron emission tomography (PET)-CT results.

## Data availability statement

The original contributions presented in the study are included in the article/supplementary material. Further inquiries can be directed to the corresponding authors.

## Ethics statement

Written informed consent was obtained from the individual(s) for the publication of any potentially identifiable images or data included in this article.

## Author contributions

LF and GZ had the original idea for the article and guided treatment and management of the patient. GH and HD did endoscopy of this patient and agreed to be accountable for any questions about endoscopic work. YL, RZ, XL, and HL collected clinical laboratory data of the patient. YZ was responsible for all pathological work in this case and ensured that questions related to the accuracy or integrity of pathological staining were appropriately investigated and resolved. LF and YL wrote the article. All authors reviewed and approved the final draft of the article.
